# Traceless Bioresponsive Shielding of Adenovirus Hexon with HPMA Copolymers Maintains Transduction Capacity In Vitro and In Vivo

**DOI:** 10.1371/journal.pone.0082716

**Published:** 2014-01-27

**Authors:** Jan-Michael Prill, Vladimír Šubr, Noemi Pasquarelli, Tatjana Engler, Andrea Hoffmeister, Stefan Kochanek, Karel Ulbrich, Florian Kreppel

**Affiliations:** 1 Department of Gene Therapy, Ulm University, Ulm, Germany; 2 Department of Biomedicinal Polymers, Institute of Macromolecular Chemistry, v.v.i., Academy of Sciences, Prague, Czech Republic; University Claude Bernard Lyon 1, France

## Abstract

Capsid surface shielding of adenovirus vectors with synthetic polymers is an emerging technology to reduce unwanted interactions of the vector particles with cellular and non-cellular host components. While it has been shown that attachment of shielding polymers allows prevention of undesired interactions, it has become evident that a shield which is covalently attached to the vector surface can negatively affect gene transfer efficiency. Reasons are not only a limited receptor-binding ability of the shielded vectors but also a disturbance of intracellular trafficking processes, the latter depending on the interaction of the vector surface with the cellular transport machinery. A solution might be the development of bioresponsive shields that are stably maintained outside the host cell but released upon cell entry to allow for efficient gene delivery to the nucleus. Here we provide a systematic comparison of irreversible versus bioresponsive shields based on synthetic *N*-(2-hydroxypropyl)methacrylamide (HPMA) copolymers. In addition, the chemical strategy used for generation of the shield allowed for a traceless bioresponsive shielding, i.e., polymers could be released from the vector particles without leaving residual linker residues. Our data demonstrated that only a bioresponsive shield maintained the high gene transfer efficiency of adenovirus vectors both in vitro and in vivo. As an example for bioresponsive HPMA copolymer release, we analyzed the in vivo gene transfer in the liver. We demonstrated that both the copolymer's charge and the mode of shielding (irreversible versus traceless bioresponsive) profoundly affected liver gene transfer and that traceless bioresponsive shielding with positively charged HPMA copolymers mediated FX independent transduction of hepatocytes. In addition, we demonstrated that shielding with HPMA copolymers can mediate a prolonged blood circulation of vector particles in mice. Our results have significant implications for the future design of polymer-shielded Ad and provide a deeper insight into the interaction of shielded adenovirus vector particles with the host after systemic delivery.

## Introduction

A large proportion of gene therapy clinical trials have been based on the use of adenovirus (Ad) gene therapy vectors [1]. Frequently, expectations have not been fulfilled in these studies and the interaction of Ad with multiple cellular and non-cellular blood components has been recognized as one of the main barriers for future clinical translation [2]–[4]. In vitro, human Ad species A, C, D, E and F (not B) infect their target cells by binding to the primary receptor CAR via the fiber knob. Subsequent binding of the penton-base-RGD to integrins stimulates shedding of fibers and endocytosis through clathrin coated pits [5]–[7]. However, in vivo it has been shown that ablation of CAR- and integrin-binding does not affect liver transduction by Ad after intravascular (i.v.) injection [8]. Rather it is the vitamin K-dependent blood coagulation factor X (FX) that, after binding to a depression in the hexon-trimer, mediates entry into hepatocytes via heparan sulfate proteoglycans (HSPGs) independent of CAR and integrins [2], [3], [9]–[11]. Apart from its strong hepatocyte tropism Ad has been shown to cause transient thrombocytopenia, interacts with other coagulation factors (i.e. FIX [2]) and is opsonized by antibodies and rapidly cleared from the circulation by liver sinusoidal endothelial cells and Kupffer cells [12]–[18].

An emerging technology to reduce undesired vector-host interactions as they occur in blood is the covalent chemical shielding of the vector surface with polymers. This strategy commonly targets 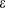-amine groups from lysine residues that are randomly distributed over the whole vector surface [19]. Covalent shielding of these amine groups with shielding polymers has already yielded promising results, including liver detargeting [20], evasion from neutralizing antibodies [21], extended plasma circulation [22] or the prevention of binding to blood components [23] (reviewed in Kreppel *et al.* [19]).

However, random shielding of the whole surface has been associated with a decreased particle infectivity [20], [23]. Therefore, defined shielding of relevant capsid positions only, combined with a traceless bioresponsive mode of polymer attachment may circumvent some of the unfavourable consequences associated with random and irreversible surface shielding. Random bioresponsive shielding of the surface of Ad has been performed using amine group-reactive PEG containing hydrazone- (pH-sensitive) or disulfide- (sensitive to reduction) containing linkers [24]. Also, amine reactive *N*-(2-hydroxypropyl)methacrylamide (HPMA) copolymers with disulfide group-containing linkers have been used [23]. So far, however, bioresponsive shielding has targeted only amine groups and invariably, after release of the polymer, residual linker groups were left behind at the site of attachment which could increase particle immunogenicity. Also amine group-directed polymer shielding modifies the whole vector surface randomly and does not allow for capsomere-specific shielding.

We use a combination of genetic and chemical (geneti-chemical) capsid modifications for vector surface shielding. We genetically introduced a cysteine residue in the hyper-variable-region 5 (HVR5) of the hexon capsomere that allows for capsomere-specific attachment of a variety of thiol group-reactive compounds including synthetic shielding polymers. Furthermore, thiol groups allow for both irreversible attachment of shielding moieties via thioether bonds and reversible attachment via disulfide bonds. The cysteine-bearing vector was called AdHexCys. Table 1 gives an overview over the total and infectious particle titers of different AdHexCys preparations.

**Table 1:** Total and infectious titers of vector preparation.

vector preparation #	total VP titer	infectious VP titer	inverse bioactivity
1	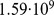	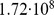	9.24
2	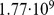	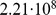	8.01
3	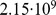	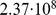	9.07

Abbreviations: VP: vector particle, titers given in VPs per 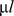.

Here we report for the first time that capsomere-specific and traceless bioresponsive shielding of the Ad capsomere hexon is feasible. Bioresponsive groups in vector systems have long been successfully used in the field of non-viral vectors as reviewed by Wolff and Rozema [25]. Non-viral vectors can be tailored using pH-responsive, enzyme cleavable or reducible groups. To shield Ad in a bioresponsive manner we activated HPMA copolymers with disulfide-based activation groups since their use is compatible with biological systems and allows for a traceless removal of the copolymer by reduction. Disulfide bonds have been shown to be sufficiently stable outside of cells while being reducible inside cells.

We synthesized a range of HPMA copolymers differing in reactive groups. HPMA copolymers were either maleimide (mal)-activated for irreversible shielding or pyridyl-dithio (PySS)-activated for traceless bioresponsive shielding. We performed a detailed in vitro and in vivo analysis of the effects of Ad shielding with these HPMA copolymers. We contrast differences that arose from the distinct modes of attachment (irreversible versus traceless bioresponsive) of otherwise similar HPMA copolymers. Our results indicate that the bioresponsive attachment is advantageous to maintain the transduction capacity of Ad while providing efficient shielding.

## Results

### HPMA copolymer synthesis and structure

**Figure 1:**
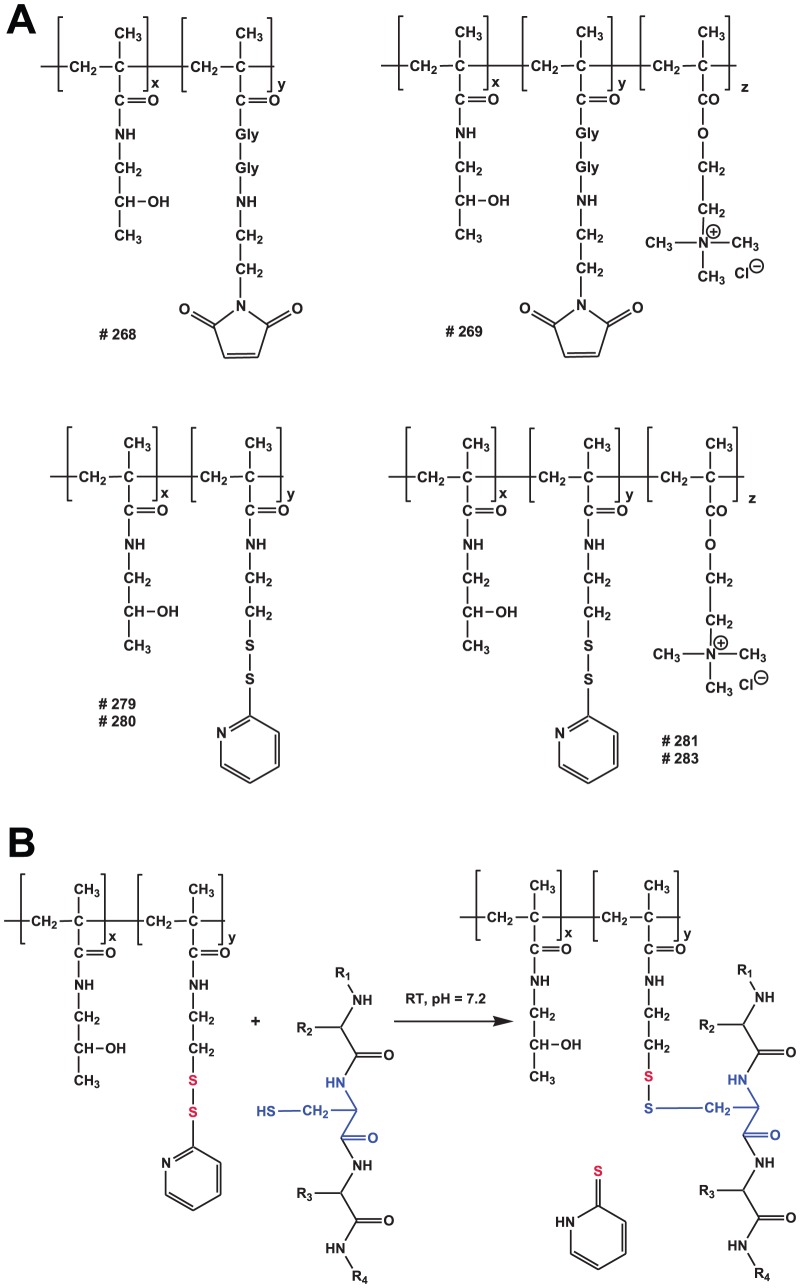
HPMA copolymer structure and bioresponsive shielding of Ad. A: Overview over the chemical structures of the HPMA copolymers. HPMA copolymers were activated with either maleimide (mal) groups or pyridyl-dithio (PySS) groups. They were either uncharged (neutral) or were positively charged via a quaternary ammonio groups. B: Reaction of a PySS-activated HPMA copolymer with the thiol group of a cysteine resulting in a traceless bioresponsive shielding of the Ad vector surface. Note the differently colored sulfur atoms indicating the thiol-exchange reaction.

The HPMA copolymers containing in the side chain maleimide or pyridyl-dithio groups designed for modification of AdHexCys via irreversible thioether bond or bioresponsive disulfide bond were synthesized by radical solution polymerization and characterized. The shielding properties of uncharged HPMA copolymers were compared with positively charged HPMA copolymers containing in the side chain besides above mentioned groups also quarternary ammonio groups. The structures of HPMA copolymers are shown in figure 1 A and characteristics are summarized in table 2. Bioresponsive shielding of AdHexCys with a PySS-activated HPMA copolymer is shown in figure 1 B.

**Table 2:** List of HPMA copolymer derivatives.

polymer #	mal [mol%]	PySS [mol%]	QA [mol%]	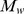 [Da]	polydispersity
268	3.55	/	/	36,800	1.94
269	2.90	/	3.00	39,800	2.21
279	/	4.10	/	40,000	1.80
280	/	8.90	/	43,200	1.80
281	/	8.50	3.00	48,000	2.47
283	/	4.66	3.00	47,900	2.47

Abbreviations: mal: maleimide content in mol%, PySS: pyridyl-dithio content in mol%, QA: quarternary ammonio group content in mol%, 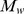: weight average molecular weight, Da: dalton.

### Western blot analysis demonstrated feasibility and bioresponsiveness of shielding with HPMA copolymers

**Figure 2:**
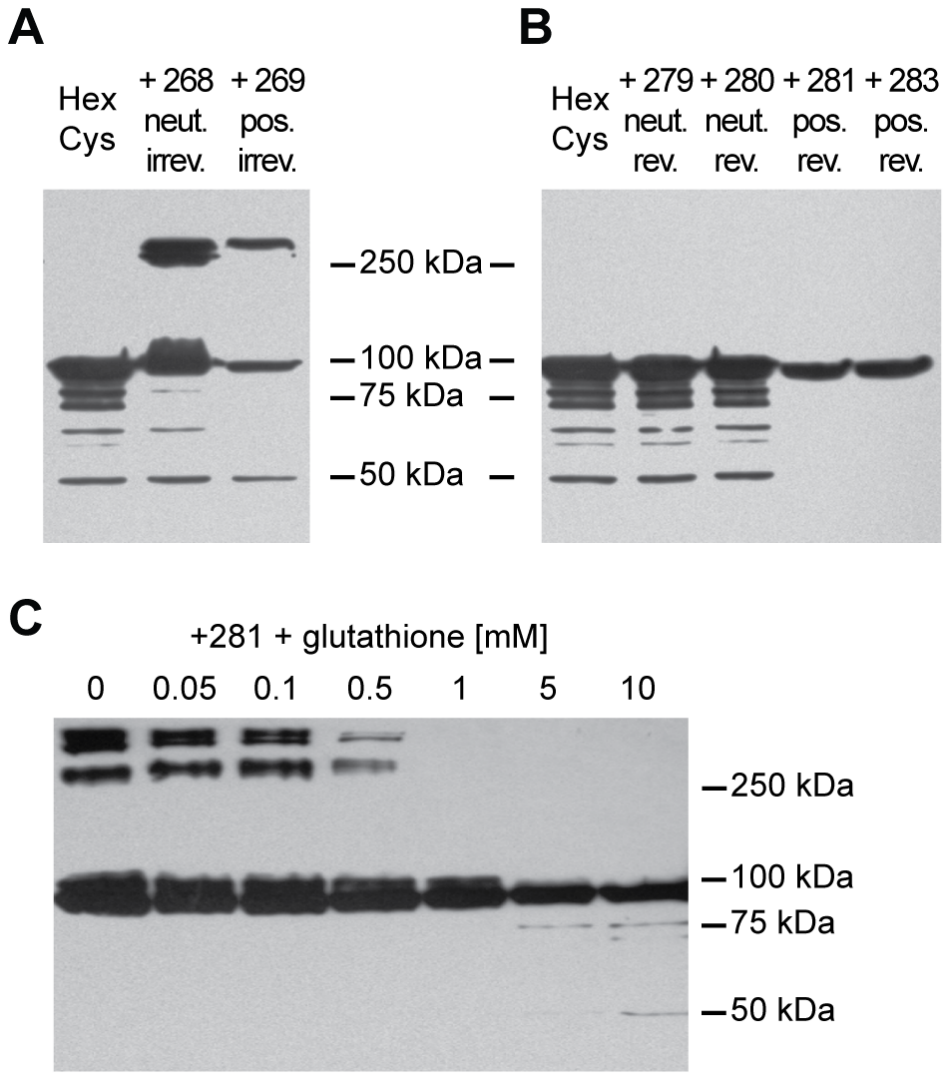
HPMA copolymers can be released by glutathione. 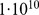
 vector particles were shielded with HPMA copolymers and analyzed by Western blot analysis using a monoclonal anti-hexon antibody. A: Ad was irreversibly shielded with HPMA copolymers # 268 or # 269. Loading buffer contained 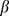-mercaptoethanol. B: Ad was shielded with the PySS-activated HPMA copolymers # 279, # 280, # 281 and # 283. Loading buffer contained 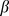-mercaptoethanol. C: Ad was shielded with the PySS-activated HPMA copolymer # 281. Before loading, the samples were pre-incubated with different concentrations of glutathione (as indicated) for 30 min at 37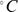. Loading buffer did not contain 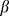-mercaptoethanol. Corresponding Western blot analyses with the HPMA copolymers # 279, # 280 and # 283 are shown in figure S1. Abbreviations: HexCys: unshielded AdHexCys, the “+ Polymer-number” indicates a shielding of AdHexCys with the respective HPMA copolymer, neut.: neutral (uncharged), pos.: positively charged, irrev.: irreversible (mal-)shielding, rev.: bioresponsive (PySS-)shielding.

To test whether shielding of AdHexCys with thiol group-reactive HPMA copolymers was feasible we analyzed 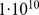 HPMA copolymer-shielded vector particles by Western blot analysis using a monoclonal anti-hexon antibody (figure 2). Shielding with mal-activated copolymers (# 268, # 269) was feasible and led to a shielding of 50% of hexon monomers (as determined by 2D-densitometry with Aida 3.12 software, figure 2 A). Since the Western blot loading buffer contained 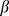-mercaptoethanol this indicated that shielding with mal-activated HPMA copolymers was irreversible and not sensitive to reduction. Shielding with PySS-activated copolymers (# 279, # 280, # 281, # 283) was feasible as well. However, in contrast to shielding with mal-activated copolymers, it was sensitive to reduction (figure 2 B). After loading with a loading buffer that contained 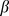-mercaptoethanol there was a quantitative release of the copolymer from hexon, showing chemoresponsiveness of the shield.

As the high concentration of 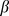-mercaptoethanol is not physiologically relevant we attempted to release the HPMA copolymer shield by glutathione, a cellular reducing agent. We preincubated the samples with different concentrations of glutathione at 37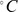 for 30 minutes followed by a semi-native Western blot analysis using loading buffer that did not contain 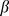-mercaptoethanol. The intracellular level of glutathione in mammalian cells is in the millimolar range (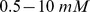) with hepatocytes having up to 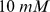 of glutathione in the cytosol [26], [27]. Importantly, after shielding with the HPMA copolymer # 281, the copolymer shield was released from the capsid in the presence of glutathione. Preincubation of the samples with glutathione led to a marked release of the copolymer at a glutathione concentration of 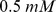 and a complete release at 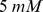 (figure 2 C). Similar results were obtained with the HPMA copolmyers # 279, # 280 and # 283 (figure S1). This strongly suggested that PySS-activated copolymers could also be released from the vector capsid inside mammalian cells. Therefore, they are not only chemoresponsive (reduction by 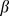-mercaptoethanol) but likely also bioresponsive (reduction by glutathione).

### Traceless bioresponsive shielding did not affect vector particle infectivity on A549 cells

**Figure 3:**
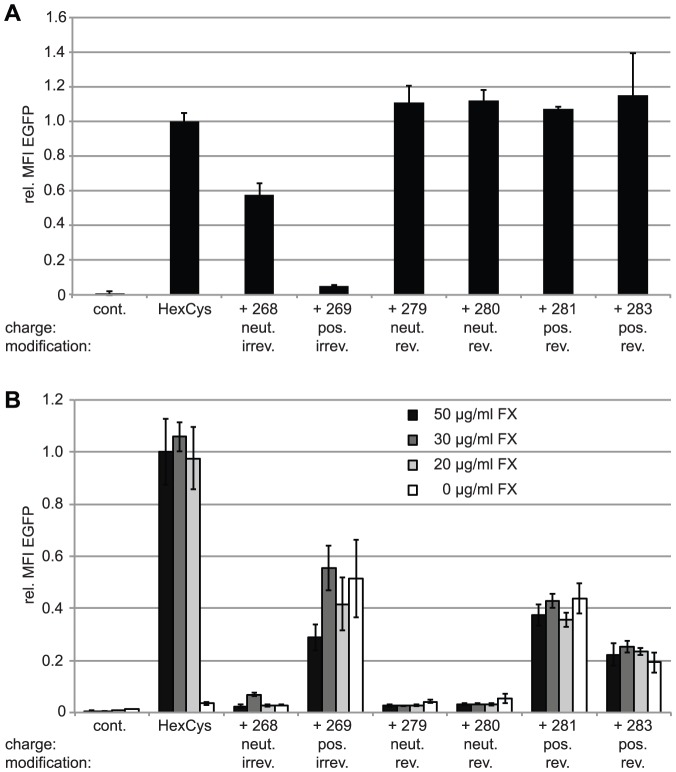
Shielding with bioresponsive HPMA copolymers maintained particle infectivity but reduced FX-mediated transduction. A: Flow-cytometric analysis of A549 cells 24 h after transduction with 200 pMOI of EGFP-expressing Ad vectors. B: Flow-cytometric analysis of SKOV-3 cells 72 h after infection with 10,000 pMOI of EGFP-expressing Ad vectors in the presence of different concentrations of FX as indicated. Note the grey scale indicating the titration of FX with the white bars indicating absence of FX. The mean fluorescence intensity of unshielded AdHexCys was set to “1” (in B: supplemented with 50 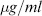 FX) and used to calculate the relative transduction efficiencies of the shielded vector particles. Abbreviations: MFI: mean fluorescence intensity, cont.: untreated control, HexCys: unshielded AdHexCys, the “+ Polymer-number” indicates a shielding of AdHexCys with the respective HPMA copolymer, neut.: neutral (uncharged), pos.: positively charged, irrev.: irreversible (mal-)shielding, rev.: bioresponsive (PySS-) shielding.

To analyze the potential bioresponsiveness of PySS-activated HPMA copolymers in vitro, A549 cells were infected with 200 pMOI of EGFP-expressing vector particles and analyzed for their EGFP expression by flow-cytometric analysis 24 h later (figure 3 A). Irreversible shielding with mal-activated copolymers decreased EGFP expression. The uncharged copolymer (# 268) decreased the EGFP expression to about 60% of the unshielded control. The positively charged copolymer (# 269) decreased the EGFP expression to about 10% of unshielded AdHexCys. In contrast, bioresponsive shielding did not affect the EGFP expression after 24 h pointing out a difference in the biological effects of the copolymers resulting from different activation groups.

### Shielding with positively charged HPMA copolymers mediated FX-independent transduction of SKOV-3 cells

In vivo hepatocyte transduction is mediated by the blood coagulation factor X (FX) which binds to hexon [3], [9]. SKOV-3 cells are used as an in vitro model for this FX-mediated transduction of hepatocytes in vivo [3], [9], [23], [28]. They are refractory to transduction by Ad in the absence of FX while in the presence of FX the transduction efficiency increases significantly. The binding sites of FX to hexon have been described in detail and are in close proximity to the cysteine residue we introduced in HVR5 [10], [29]. To analyze whether shielding with HPMA copolymers prevented FX-mediated transduction we used the SKOV-3 cell model. Cells were transduced with 10,000 pMOI of EGFP expressing vector particles in the presence of 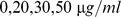 FX and analyzed for their EGFP expression by flow cytometric analysis 72 h later (figure 3 B). Independent of the HPMA copolymer used for shielding, all copolymers mediated a decrease in EGFP expression. Uncharged HPMA copolymers (# 268, # 279, # 280) reduced the EGFP expression to control level independent of the FX concentration present. In contrast, positively charged HPMA copolymers (# 269, # 281, # 283) mediated transduction of SKOV-3 cells even in the absence of FX (note the grey scale indicating the FX-titration with the white bars indicating absence of FX). This transduction was not increased even in the presence of supraphysiological concentrations of FX (physiologic concentration is 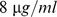) demonstrating efficient abrogation of FX-mediated effects by the HPMA copolymers (figure 3 B). By comparison with the uncharged HPMA copolymers which are similar except for their charge, it can be concluded that the transduction of SKOV-3 cells was dependent only on the positive charge of the copolymer itself but independent of FX.

### Shielding with HPMA copolymers did not affect entry into cells but did affect intracellular trafficking

**Figure 4:**
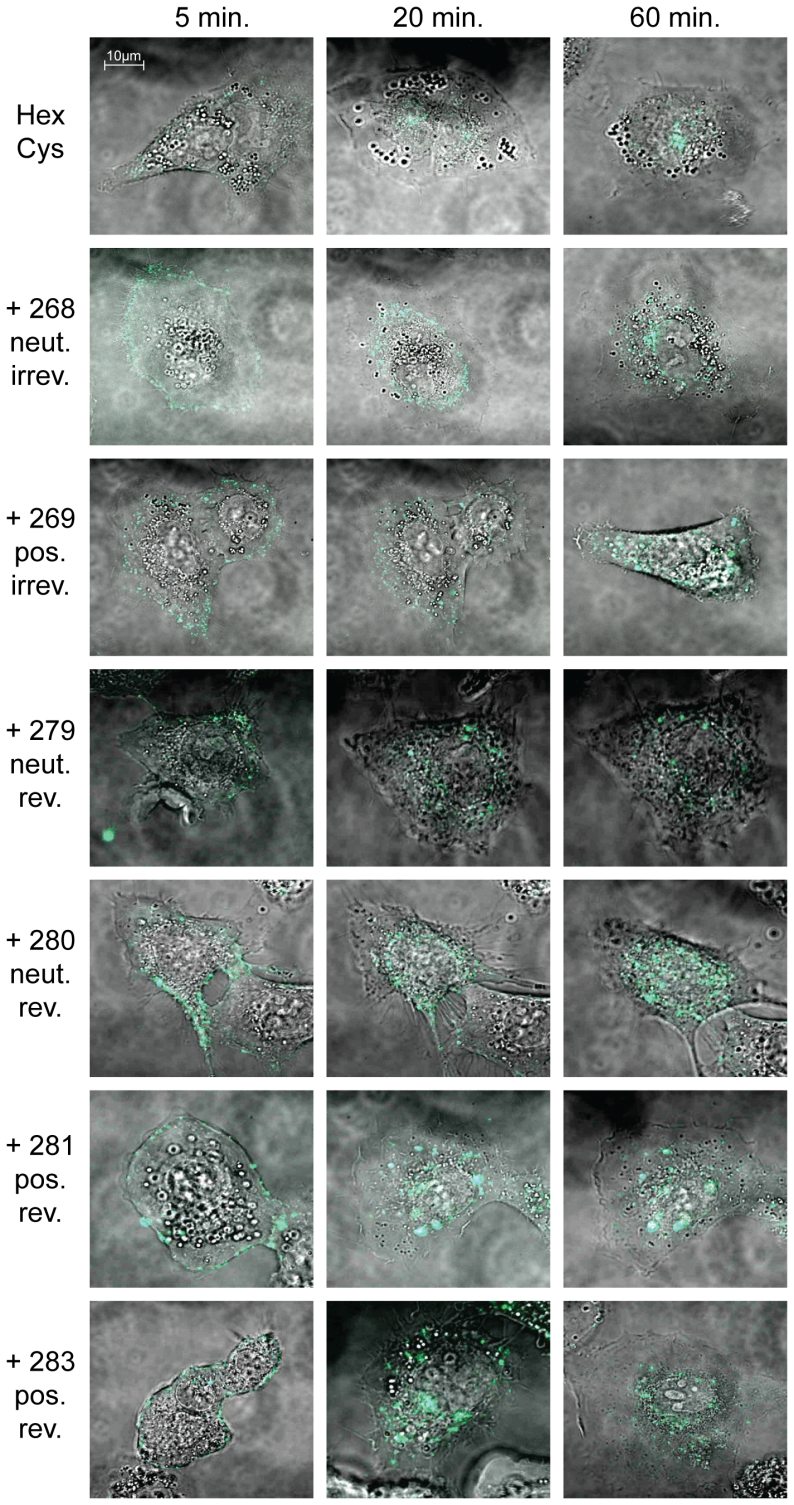
Bioresponsive shielding resolved intracellular trafficking impairment of shielded Ad particles. A549 cells were transduced with 10,000 pMOI of Alexa-488 stained HPMA copolymer shielded vector particles. Particle entry into and trafficking inside A549 cells was visualized using confocal laser scanning microscopy. It was reported before that AdHexCys shows wild type like particle trafficking [24]. HPMA copolymer shielding did not affect particle entry into A549 cells. Irreversible shielding mediated a trafficking impairment. Bioresponsive shielding mediated a trafficking delay. Not all particles had reached the nuclear membrane at the end of the observed time. However, it can be assumed that eventually all particles will reach the nucleus since no difference in EGFP expression was observed after 24 h for these samples (see flow cytometric analysis in figure 3 A). Note that within each sample different representative pictures are shown. Abbreviations: HexCys: unshielded AdHexCys, the “+ Polymer-number” indicates a shielding of AdHexCys with the respective HPMA copolymer, neut.: neutral (uncharged), pos.: positively charged, irrev.: irreversible (mal-)shielding, rev.: bioresponsive (PySS-)shielding.

To visualize particle entry into and trafficking inside cells, A549 cells were transduced with Alexa-488 stained vector particles and were analyzed by live cell confocal microscopy (figure 4). Unshielded Alexa-stained vector particles had entered the cells after 5 minutes and the majority of the particles had reached the nuclear membrane after 20 minutes, indicating that Alexa-modification did not affect regular particle entry and trafficking. Wild-type like particle trafficking of AdHexCys particles has been demonstrated earlier [24]. Independent of the HPMA copolymer used, all shielded particles had entered the cells after 5 minutes. However, intracellular trafficking was affected by HPMA copolymer-shielding with both the activation group and the charge of the copolymer influencing it to different degrees. Irreversible shielding with mal-activated HPMA copolymers mediated an impairment in nuclear trafficking. This was especially prominent when the positively charged copolymer (# 269) was used for shielding. In contrast, bioresponsive shielding with PySS-activated copolymers allowed for particle trafficking to the nucleus for which we observed a slight delay. During the time of observation (1 h) many, yet not all bioresponsively shielded particles had reached the nucleus (figure 4).

In summary, our in vitro data indicated that PySS-activated copolymers were traceless bioresponsive and were released from the vector particle after entry into A549 cells. Shielding with HPMA copolymers appeared to abolish FX-mediated transduction of SKOV-3 cells. Positively charged HPMA copolymers mediated FX independent transduction of SKOV-3 cells.

### Traceless bioresponsive shielding with positively charged HPMA copolymers mediated hepatocyte transduction

**Figure 5:**
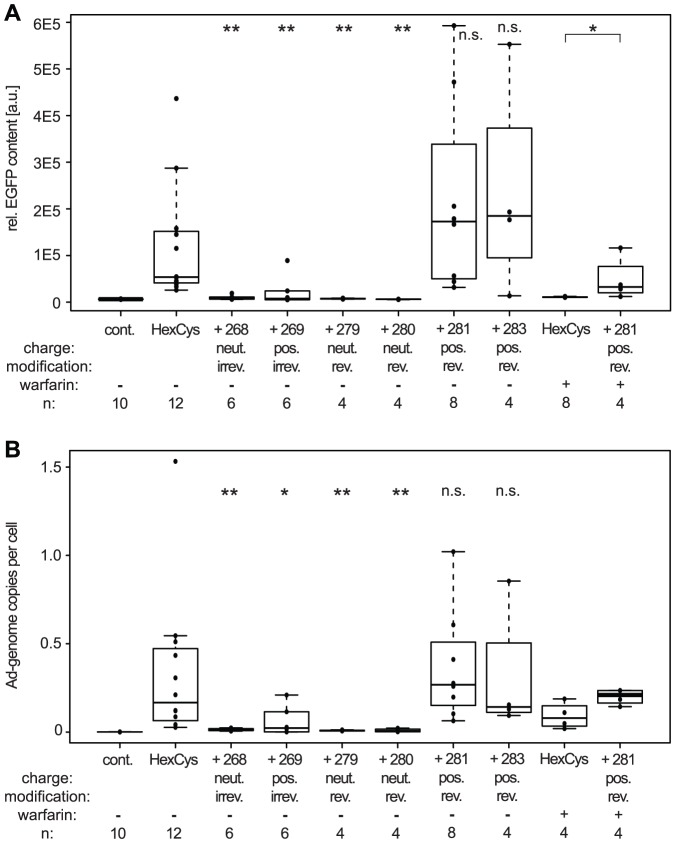
Positively charged bioresponsive HPMA copolymers mediated robust transduction of liver. Female BALB/c mice were injected with 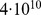 HPMA copolymer-shielded EGFP-expressing Ad vector particles. 72 h later livers were harvested and processed for fluorimetry (A) and QPCR analysis (B). A: A part of shock frozen liver was homogenized and the relative EGFP expression was analyzed by fluorimetry. B: DNA was isolated from a part of shock frozen liver and was analyzed for its Ad-genome content by QPCR. Abbreviations: cont.: untreated control, HexCys: unshielded AdHexCys, the “+ Polymer-number” indicates a shielding of AdHexCys with the respective HPMA copolymer, neut.: neutral (uncharged), pos.: positively charged, irrev.: irreversible (mal-)shielding, rev.: bioresponsive (PySS-)shielding, n.s.: not significant, p<0.05 (*), p<0.005 (**), significance levels referring to unshielded AdHexCys except when indicated differently.

**Figure 6:**
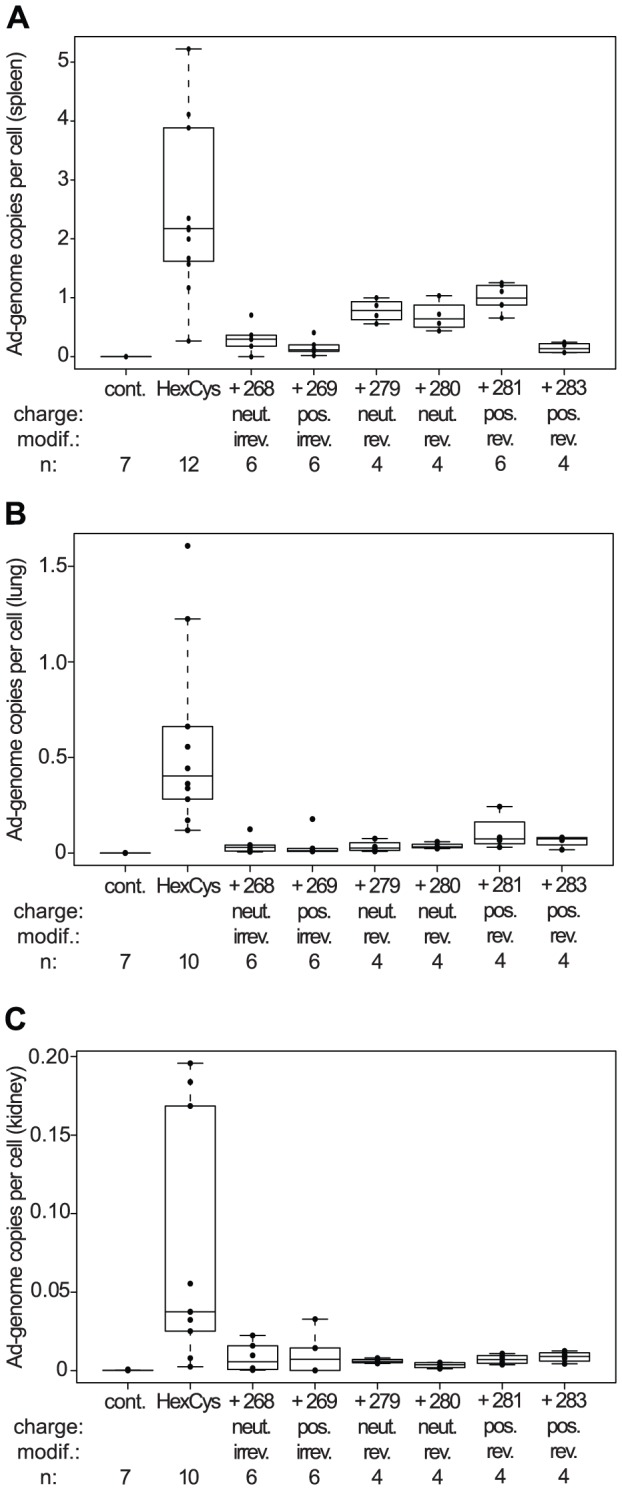
HPMA copolymer shielding reduced vector promiscuity. Female BALB/c were injected with 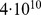 HPMA copolymer-shielded EGFP-expressing Ad vector particles. 72 h later organs were harvested and processed for QPCR analysis. Independent of the HPMA copolymer used, a marked reduction in the Ad genome level was detected in the spleen (A) the lung (B) and the kidney (C) compared to unshielded AdHexCys. Abbreviations: cont.: untreated control, HexCys: unshielded AdHexCys, the “+ Polymer-number” indicates a shielding of AdHexCys with the respective HPMA copolymer, neut.: neutral (uncharged), pos.: positively charged, irrev.: irreversible (mal-)shielding, rev.: bioresponsive (PySS-)shielding.

**Figure 7:**
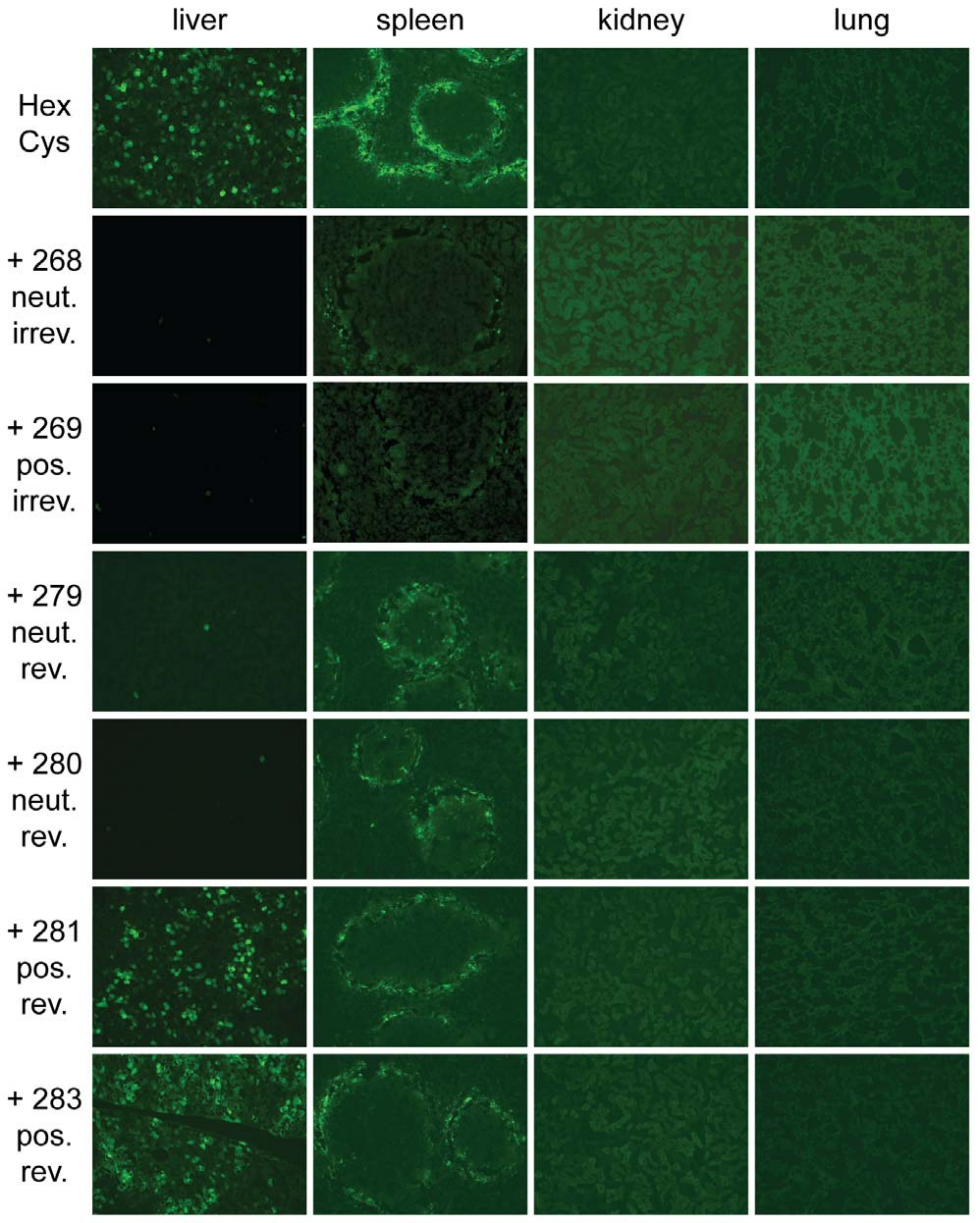
Histological cryosections: reduced EGFP expression in the spleen after shielding of Ad with HPMA copolymers. Female BALB/c were injected with 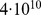 HPMA copolymer-shielded EGFP-expressing Ad vector particles. 72 h later organs were harvested and analyzed by histological cryosections. Magnification: 100-fold, exposure time: liver 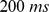, spleen 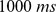, kidney 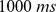, lung 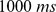, Abbreviations: HexCys: unshielded AdHexCys, the “+ Polymer-number” indicates a shielding of AdHexCys with the respective HPMA copolymer, neut.: neutral (uncharged), pos.: positively charged, irrev.: irreversible (mal-)shielding, rev.: bioresponsive (PySS-)shielding.

**Figure 8:**
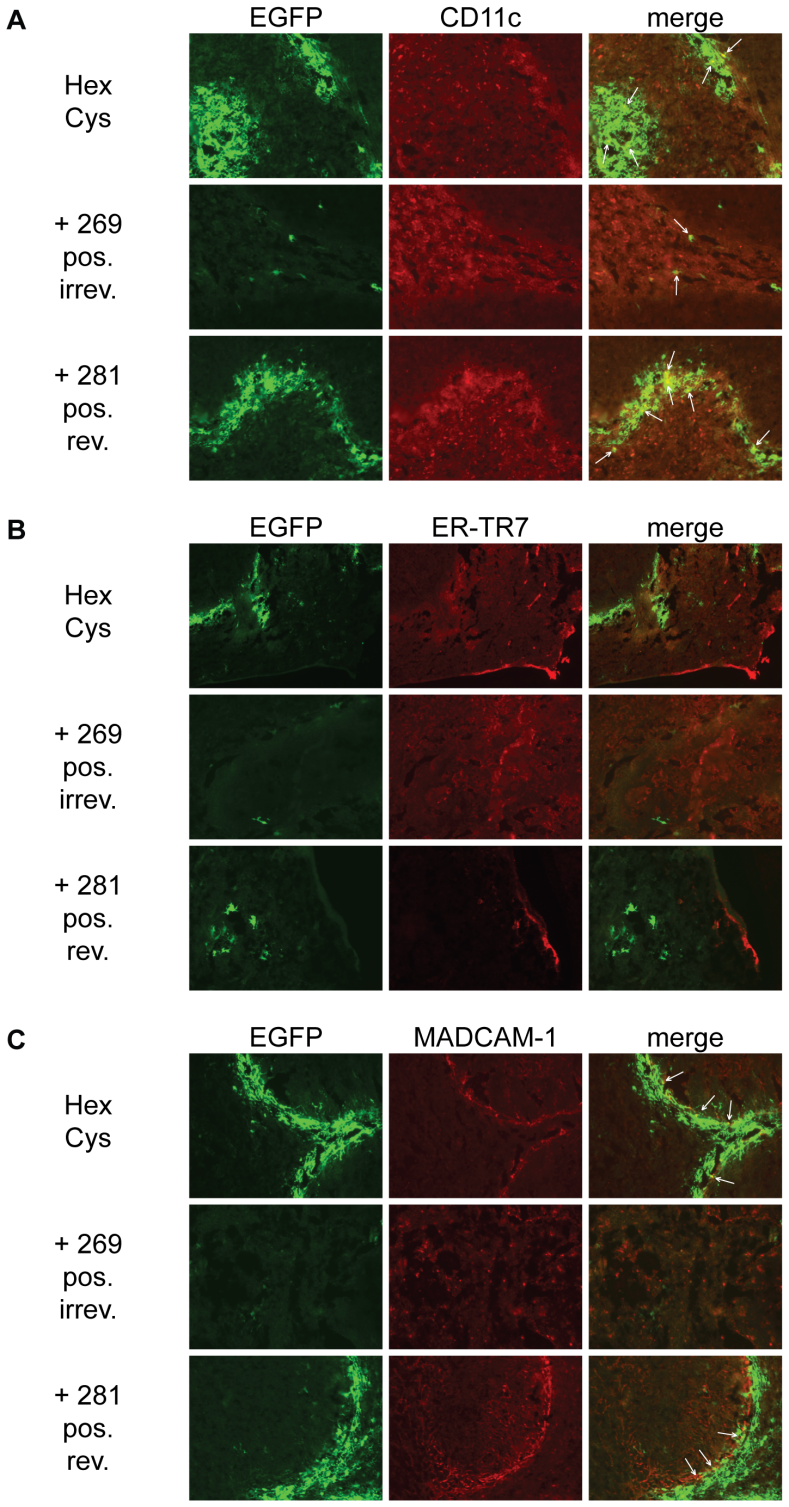
HPMA copolymer shielding did not alter the transduction pattern in the spleen. To analyze whether HPMA copolymer shielding altered the cell type in the spleen that was transduced after vector injection we performed immunohistological staining of histological cryosections against CD11c (A), ER-TR7 (B) and MADCAM-1 (C). Female BALB/c were injected with 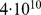 HPMA copolymer-shielded EGFP-expressing Ad vector particles. 72 h later spleens were harvested and analyzed by histological cryosections. Representative sections are shown. Several cell types in the spleen appear to be transduced by Ad mainly in the marginal zone. Shielding with HPMA copolymers reduced the overall transduction but did not seem to alter the transduction pattern or type of transduced cell. Representative sections are shown. HPMA copolymers # 268, # 279, # 280 and # 283 not shown. Abbreviations: HexCys: unshielded AdHexCys, the “+ Polymer-number” indicates a shielding of AdHexCys with the respective HPMA copolymer, neut.: neutral (uncharged), pos.: positively charged, irrev.: irreversible (mal-)shielding, rev.: bioresponsive (PySS-)shielding.

To analyze potential in vivo differences that arise from the distinct ways of polymer attachment of otherwise similar HPMA copolymers and to evaluate the potential of positively charged HPMA copolymers to mediate hepatocyte transduction, 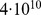 EGFP-expressing vector particles were injected into the tail vein of 6–8 week old female BALB/c mice in a total volume of 200 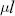. 72 h later the organs were harvested and processed for fluorimetric analysis (figure 5 A), quantitative PCR (QPCR; figures 5 B and 6), histological cryosections (figure 7) and immunohistological analysis of the spleen (figure 8).

Irreversible shielding with mal-activated HPMA copolymers (# 268, # 269) or bioresponsive shielding with uncharged PySS-activated HPMA copolymers (# 279, # 280) abolished EGFP expression in liver almost completely (figure 5 A). In contrast, bioresponsive shielding with positively charged PySS-activated HPMA copolymers (# 281, # 283) mediated liver transduction similar to that obtained with unshielded AdHexCys.

Our in vitro data indicated that positively charged HPMA copolymers might mediate transduction independent of FX. To analyze this effect in vivo we depleted mice of vitamin K-dependent coagulation factors (including FX) by warfarin treatment. Warfarin is an anticoagulant which blocks the recycling of active vitamin K, thereby depleting the vitamin K-dependent coagulation factors [30]. Treatment of mice with warfarin is commonly used to abolish coagulation factor-mediated liver transduction [2], [28], [31]. Consequently, treatment of mice with warfarin before the injection of unshielded AdHexCys abolished hepatocyte transduction almost completely (figure 5 A). However, after injection of vector shielded with the HPMA copolymer # 281 (traceless bioresponsive and positively charged) into warfarin treated mice, hepatocyte transduction was partially restored. Shielding with the HPMA copolymer # 281 mediated a significant increase in EGFP expression in the liver compared to unshielded AdHexCys (figure 5 A).

QPCR analysis of liver DNA corroborated the fluorimetric analysis and revealed that shielding with uncharged HPMA copolymers (# 268, # 279, # 280) significantly reduced the Ad genome level to control level (figure 5 B). After shielding with positively charged HPMA copolymers (# 269, # 281, # 283) higher amounts of Ad genomes were detected compared to their uncharged counterparts. After bioresponsive shielding with positively charged PySS-activated HPMA copolymers (# 281, # 283) Ad genome levels detected were significantly higher compared to their uncharged equivalents (p<0.05, not indicated in the figure) and there was no significant difference to unshielded AdHexCys (figure 5 B).

### HPMA copolymer shielding reduced vector promiscuity

Ad biodistribution was analyzed in detail by QPCR analysis of liver DNA (figure 5 B), spleen, lung and kidney DNA (figures 6 A–C, respectively) and by histological cryosections (figure 7). QPCR analysis of spleen, lung and kidney revealed a decrease in the Ad genome copy number after shielding independent of the HPMA copolymer used. We did not observe significant differences independent of the charge of the HPMA copolymer (neutral versus positive) or the way of copolymer attachment (irreversible versus traceless bioresponsive).

### Histological cryosections: Reduced EGFP expression in the spleen after shielding of Ad with HPMA copolymers

Systemic delivery of unshielded AdHexCys mediates robust and wild type like transduction of the liver and the spleen [28] with the latter showing the typical transduction pattern mainly in the marginal zone (figure 7). After injection of Ad vectors that were irreversibly shielded (HPMA copolymers # 268, # 269) only very few, weakly EGFP-expressing cells were found in the liver and the spleen independent of the copolymer charge (figure 7). However, after injection of bioresponsively shielded Ad vectors EGFP expression strongly depended on the charge of the HPMA copolymer, corroborating the fluorimetric analysis in figure 5 A. Uncharged bioresponsive HPMA copolymers (# 279, # 280) mediated a similarly weak liver transduction as did the irreversibly shielding copolymers. However, positively charged bioresponsive copolymers (# 281, # 283) mediated a liver transduction that was not different from that obtained after injection of unshielded AdHexCys. In contrast to liver, transduction of spleen was independent of the copolymer charge when bioresponsive HPMA copolymers were used for shielding. Bioresponsive copolymers reduced the EGFP expression in the marginal zone of the spleen compared to unshielded AdHexCys. Yet, they mediated a stronger EGFP expression compared to the irreversibly attached copolymers which abolished EGFP expression almost completely. In the kidney and lung no EGFP expression was observed after injection of unshielded AdHexCys or any other shielded Ad vector independent of its shield (figure 7).

In order to further analyze whether shielding with HPMA copolymers affected the transduced cell type in the spleen we performed immunohistological analysis of spleen sections.

### HPMA copolymer shielding did not alter the transduced cell type in the spleen

Upon systemic delivery of Ad the spleen is transduced mainly in the marginal zone. Immunohistological staining of transgene positive cells suggested that not a single cell type but a number of different cell types are transduced by Ad [12], [32]. To analyze whether shielding with HPMA copolymers had effects on the cell type that was transduced, spleen sections were analyzed by immunohistological staining 72 hours after systemic delivery of shielded Ad vectors (figure 8 A–C). Spleen sections were stained against CD11c (A), reticular fibroblasts and reticular fibres (ER-TR7; B) and mucosal vascular addressin cell adhesion molecule 1 (MADCAM-1; C). CD11c is found in high levels on dendritic cells which have been shown to be transduced by Ad after systemic vector delivery. Independent of the HPMA copolymer shield some co-localizing EGFP^+^ and CD11c^+^ cells were found. Shielding with HPMA copolymers did not appear to alter the distribution of transduced cells. ER-TR7 and MADCAM-1 have been reported to partially co-localize with transgene expressing cells after systemic delivery of Ad [32]. We did not observe co-localization of EGFP^+^ and ER-TR7^+^ cells. For MADCAM-1^+^ cells about the same degree of co-localization as for CD11c^+^ cells was observed. Overall, as reported before, several cell types located in the marginal zone of the spleen appearently were transduced by Ad (figure 8). Shielding with HPMA copolymers reduced the overall transduction but did not alter the transduction pattern or type of transduced cell.

### Shielding with HPMA copolymers mediated prolonged blood circulation of AdHexCys

**Figure 9:**
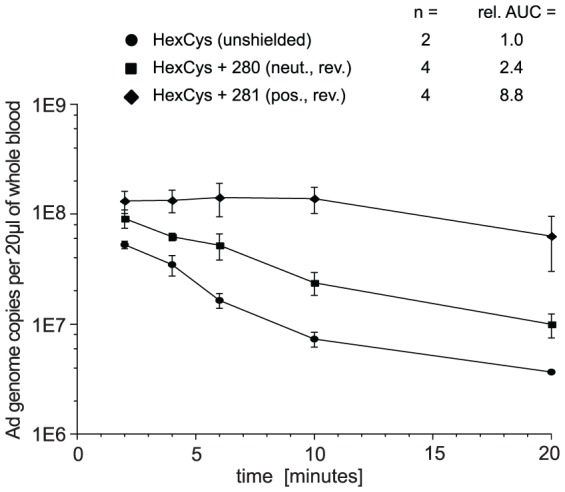
Blood circulation kinetics of HPMA copolymer shielded AdHexCys. To analyze whether shielding with HPMA copolymers would alter the blood circulation kinetics of AdHexCys we injected BALB/c mice with 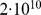 HPMA copolymer-shielded vector particles. 2, 4, 6, 10 and 20 min after vector delivery 20 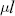 of blood were taken from the tail vein with a glass capillary by scoring. Total DNA was extracted and Ad genome content was analyzed by QPCR. HPMA copolymers # 268, # 269, # 279 and # 283 not tested. Abbreviations: HexCys: unshielded AdHexCys, the “+ Polymer-number” indicates a shielding of AdHexCys with the respective HPMA copolymer, neut.: neutral (uncharged), pos.: positively charged, rev.: bioresponsive (PySS-) shielding, rel. AUC: relative area under the curve.

Upon systemic delivery Ad is rapidly cleared from the circulation [14], [18], [22]. To evaluate whether the transduction of liver that was observed after shielding of AdHexCys with the HPMA copolymers # 281 or # 283 correlated with prolonged circulation we analyzed the blood circulation kinetics of shielded AdHexCys. 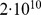 HPMA copolymer-shielded vector particles were injected in BALB/c mice in a total volume of 200 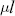. After 2, 4, 6, 10 and 20 min blood was taken and analyzed for its Ad genome content by QPCR (figure 9). Unshielded AdHexCys was rapidly cleared from the circulation, similar to published data of wild type capsid vectors [14], [22]. Shielding with the HPMA copolymer # 280 (neutral, bioresponsive) mediated an only mild 2.4-fold increase of the area under the curve compared to unshielded AdHexCys. However and surprisingly, shielding with the HPMA copolymer # 281 (positively charged, bioresponsive) mediated a 8.8-fold increase of the area under the curve compared to unshielded AdHexCys (figure 9). Of note, shielding with this copolymer also maintained liver transduction by AdHexCys (see figure 5 A).

## Discussion

An emerging technology to prevent undesired interactions of Ad with host blood and tissue is the shielding of the Ad surface with polymers. However, commonly the whole vector surface is shielded randomly or, if shielding is capsomere-specific, it has been in an irreversible manner. We report that shielding polymers can be attached both in a capsomere-specific and in a traceless bioresponsive manner, leaving no residual chemical groups on the vector surface after release of the polymer. Using PySS group-activated HPMA copolymers we demonstrated (i) that hexon-specific traceless bioresponsive shielding of Ad was feasible, (ii) that bioresponsive shielding of hexon did not affect particle infectivity in vitro and (iii) that bioresponsive shielding could resolve nuclear DNA delivery impairments often associated with irreversible shielding. As a proof of concept we showed that HPMA copolymers could mediate transduction of liver independent of FX if the copolymer was positively charged and attached in a traceless bioresponsive manner. Also, we demonstrated that shielding of AdHexCys with HPMA copolymers could prolong the blood circulation of the vector particles.

Flow cytometric analysis of A549 cells revealed that irreversible shielding reduced particle infectivity whereas bioresponsive shielding did not. This was in contrast to the results obtained by Espenlaub *et al.* where PEG linked to the vector surface via disulfide-containing linkers was shown not to be released efficiently [24]. Western blot analysis implied a 50% modification of hexon whereas about 10,000 amine residues were shielded by Espenlaub *et al.*. This demonstrated the limits in the reductive capacity of A549 cells. They were unable to reduce the many disulfide bonds after amine group shielding while being able to reduce the disulfide bonds after capsomere-specific hexon shielding with only about 360 disulfide bonds to be reduced. This is important since Fisher *et al.* obtained particles of which 70% of the amine groups were (irreversibly) shielded with amine group-directed HPMA copolymers [21]. It can be concluded that amine group-directed shielding with HPMA copolymers containing (bioresponsive) disulfide-based linker groups would suffer from the same shortcomings observed by Espenlaub *et al.* with the polymers not being released efficiently after cell entry.

On SKOV-3 cells positively charged HPMA copolymers mediated FX independent transduction. The HPMA copolymer shield efficiently prevented FX-mediated effects even in the presence of supraphysiological concentrations since the presence of FX did not increase transduction compared to the samples without FX. Positive charge-mediated transduction by Ad is in line with other reports using a poly(lysine) insertion in fiber [33] or shielding of the Ad surface with cationic polyamidoamine dendrimers [34]. In the presence of FX, Ad vectors shielded with positively charged HPMA copolymers mediated a reduced transduction compared to unshielded AdHexCys. We attribute this to a reduced negative surface charge of an Ad capsid that is shielded with positively charged HPMA copolymers compared to a FX-decorated Ad capsid.

Live cell imaging revealed a trafficking impairment of irreversibly shielded Ad particles, especially when the positively charged copolymer was used. In contrast, bioresponsively shielded particles showed only a trafficking delay. The flow cytometric analysis of A549 cells demonstrated that bioresponsive shielding of AdHexCys did not affect EGFP expression after 24 h. Hence, it appears likely that the irreversibly shielded particles suffered from an impairment in nuclear DNA delivery, whereas the bioresponsively shielded particles eventually delivered their DNA into the nucleus. Thus, these data provide evidence that traceless bioresponsive shielding can resolve trafficking impairments mediated by irreversible shielding in vitro.

Next, we performed a detailed in vivo side by side comparison of the effects of positively charged versus neutral HPMA copolymers together with a comparison of irreversible versus traceless bioresponsive HPMA copolymer shielding.

EGFP expression analysis 72 h after vector delivery revealed that there were profound differences in EGFP expression in the liver depending on the way of shielding or the charge of the HPMA copolymer. Irreversible shielding of AdHexCys abolished EGFP expression almost completely. In vitro the positively charged mal-activated HPMA copolymer (# 269, irreversible shielding) mediated similar EGFP expression on SKOV-3 cells as did the positively charged PySS-activated HPMA copolymers (traceless bioresponsive shielding). Since we did not observe transduction mediated by this HPMA copolymer in vivo, we hypothesize that this might be due to a difference in vector uptake or intracellular trafficking between the human ovary adenocarcinoma SKOV-3 cell line in vitro and the murine hepatocytes in vivo. The bioresponsively shielded particles only mediated EGFP expression in the liver when a positively charged HPMA copolymer was used which compensated for the lack of FX-mediated effects. The differences in EGFP expression mediated by irreversible shielding compared to bioresponsive shielding indirectly prove that the bioresponsive HPMA copolymers were released from the Ad capsid in vivo. This release may not have been complete but was sufficient not to interfere anymore with nuclear DNA delivery. In vivo, after release, linear HPMA copolymers of up to 70 kDa can be eliminated from the body by glomerular filtration and urinary excretion [35].

After treatment of mice with warfarin we observed that shielding with a bioresponsive positively charged HPMA copolymer (# 281) mediated a significant increase in EGFP expression in the liver compared to unshielded AdHexCys. However, warfarin treatment still significantly reduced EGFP expression in the liver mediated by this HPMA copolymer (# 281) compared to mice that were not treated with warfarin. We observed that, on SKOV-3 cells, shielding with an HPMA copolymer completely abolished FX-mediated effects and that positively charged HPMA copolymers could compensate for this and mediate transduction independent of FX via their charge. Therefore, it is remarkable that warfarin treatment reduced EGFP expression in the liver of the copolymer # 281 sample. Of note, despite warfarin treatment transduction was still feasible in contrast to the unshielded vector.

These data can be interpreted in multiple ways. It may be hypothesized that other vitamin K-dependent blood coagulation factors distinct from FX are involved in hepatocyte transduction by Ad shielded with the positively charged HPMA copolymer # 281 in vivo. Factors II, VII, IX, X and proteins C, S, and Z are dependent on vitamin K [36]. Factors VII, IX, X and protein C but not factors II, XI and XII have been shown to enhance hepatocyte transduction in vitro [2]. However, this model can probably not explain the observed residual transduction after warfarin treatment as it was mediated by the shielded but not the unshielded vectors. If transduction could only be attributed to one additional blood coagulation factor, transduction mediated by both shielded and unshielded vectors should drop to the same level (close to zero) after warfarin treatment. This was not the case, yet.

Importantly, besides bridging the vector particles to HSPGs on hepatocytes Xu *et al.* revealed another role of FX for Ad5, namely shielding of the Ad particles from IgM/complement-mediated inactivation [37]. When FX is absent (and thus does not occupy its binding sites on the Ad capsid), natural IgM can bind to the vectors and activate complement system leading to vector inactivation. After polymer coating the up to date unknown IgM binding sites on the Ad capsid may be at least partially shielded and the respective vectors remain partially active. Therefore, our data suggest that shielding against IgM binding by the HPMA polymers may be active but incomplete. This model could explain residual transduction mediated by the shielded but not the unshielded vectors after warfarin treatment. Of course, we cannot exclude that other as yet unidentified non-cellular (or even cellular) interactions of Ad with host components further tangle the interpretation. For example it may be possible that another blood coagulation factor distinct from FX is involved in the transduction of liver by Ad. Involvement of this factor has so far not become evident due to IgM-mediated inactivation of particles when FX-binding was ablated genetically or by X-bp [3], [32], [37]. Conclusively, FX appears to the central mediator of hepatocyte transduction by Ad. A similar conclusion has recently been drawn by Baker *et al.*, as well [38].

Histological cryosections of the spleen revealed a higher EGFP expression in the spleen after bioresponsive shielding compared to irreversible shielding. This indicated that also in the spleen bioresponsive HPMA copolymers were released from the Ad capsid. It has been reported by Taylor *et al.* and Khynriam *et al.* that the spleen also expresses relatively high levels of glutathione (about 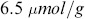 wet weight compared to liver with about 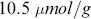) [39], [40]. This suggested the release of the bioresponsive HPMA copolymers also in the spleen. Importantly, in tumor, supraphysiological levels of glutathione are reported to be associated with chemotherapy resistance [41]. Therefore, it is likely that release of this bioresponsive HPMA copolymer will also occur in tumor tissue.

In extrahepatic tissues, shielding with HPMA copolymers mediated a significant decrease in the Ad genome copy number per cell compared to unshielded AdHexCys. Shielding with HPMA copolymers interfered with the infection of extrahepatic tissues independent of the nature of the HPMA copolymer, e.g. by an interference with intracellular trafficking or receptor binding. Transduction of liver mediated by shielding with the HPMA copolymers # 281 and # 283 was due to the special nature of transduction of the liver which is mediated by FX and other coagulation factor(s). FX-mediated effects could be mimicked by positively charged, bioresponsive HPMA copolymers.

Identification of the exact cell type in the spleen is challenging since no marker tested so far seems to be expressed on all transgene expressing cells [32]. Immunohistological analysis of sections indicated that in general several cell types in the spleen appear to be transduced by Ad mainly in the marginal zone. Shielding with HPMA copolymers reduced the overall transduction but did not alter the transduction pattern or type of transduced cells.

Importantly, we demonstrated that specific shielding of hexon with bioresponsive HPMA copolymer prolonged the blood circulation of AdHexCys. Shielding with a neutral HPMA copolymer mildly increased blood circulation of AdHexCys and shielding with a positively charged HPMA copolymer strongly enhanced blood circulation of AdHexCys. Since the most significant difference between the two HPMA copolymers was their charge, we assume that the positively charged HPMA copolymer interacts differently with the Ad capsid compared to the neutral HPMA copolymer according to Šubr *et al.* [23]. Positively charged HPMA copolymers may preferentially or more effectively shield capsid regions that are involved in particle clearance from the blood. These data corroborated our hypothesis that HPMA copolymers mediated transduction of liver based on their charge.

After circulating for 20 minutes, 17.3-fold more vector particles were detected in the blood when shielded with the HPMA copolymer # 281 compared to unshielded vector particles. Varying values for the circulating blood volume of laboratory animals can be found in literature. Assuming a blood volume of 1.46 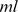 [42] to 1.80 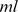 [43] for a 25 g mouse, there is a maximal vector concentration of 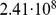 to 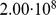 vector particles per 20 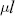 of blood after delivery of 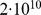 vector particles (delivered in 200 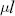 ). After shielding of AdHexCys with the HPMA copolymer # 281, 56.7% to 68.3% of the injected dose was circulating in the blood over a period of 10 minutes, having dropped to 26.3% to 31.6% after 20 minutes. Development of EPR-effect-targeted oncolytic vectors would benefit from such a prolonged circulation, especially as fiber is unmodified and could incorporate targeting ligands.

Further research will be required to analyze these vectors' potential to evade the host barriers they would face in an immunocompetent human, like the binding to erythrocytes and the binding of high affinity antibodies. It appears unlikely, however, that hexon-specific shielding will prevent all undesired interactions. Also, only a portion of anti-Ad antibodies will be shielded by hexon-specific shielding. However, for a defined shielding of only relevant capsid positions (i.e. main antibody epitopes) these would need to be characterized in detail first. Therefore, to date, it appears to be mandatory to extensively combine polymer capsid shielding at carefully chosen sites with other genetic alterations ranging from point mutations (to knock out CAR- or FX-binding [29], [32], [44]), over fiber-switching to the use of other Ad types with low seroprevalence.

We highlighted that a careful analysis of shielded vector particles, including intracellular trafficking is mandatory for the understanding of in vivo effects of the vector. We demonstrated that A549 cells can reduce 360 disulfide groups per shielded Ad particle whereas the reductive capacity is exceeded with 10,000 disulfide groups per particle [24]. Importantly, we provided evidence that the HPMA copolymers were released in vivo in hepatocytes and splenocytes. We showed that positively charged polymers could mediate hepatocyte transduction in vivo as also observed with other positively charged polymers (e.g. dendrimers) [34]. Importantly, we demonstrated that shielding with HPMA copolymers could prolong the blood circulation of AdHexCys. In summary, our study highlights the need of a thorough understanding of vector-polymer-host interactions and the requirement of a careful polymer design to avoid new and eventually undesired polymer-host interactions.

## Materials and Methods

### Plasmids and bacmids

The first-generation adenovirus serotype 5-based (Ad) vector was a derivative of pGS66, an E1-deleted infectious plasmid described previously [45]. The E1-deleted Ad genome was cloned into a bacmid based on pBeloBac11 (New England Biolabs, Ipswich, MA) and the cysteine residue in hexon HVR5 was introduced by homologous recombination using the homologous recombination system from GeneBridges (Heidelberg, Germany). The sequence of the HVR5 of AdHexCys is TTEACAGNGDNLT (wild-type HVR5: TTEAAAGNGDNLT). Subsequently, an human cytomegalovirus promoter driven expression cassette for EGFP was cloned into the E1-deleted Ad genome.

### Cell lines and cell culture

Media were supplemented with fetal bovine serum (FCS) and 1% penicillin-streptomycin-glutamine. Cells were passaged twice a week. A549 cells (ATCC No. CCL-185) were maintained in MEM (Gibco, Carlsbad, CA) with 10% FCS, N52.E6 cells [45] were maintained in 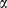-MEM (Gibco, Carlsbad, CA) with 10% FCS and SKOV-3 (ATCC No. HTB-77) cells were maintained in RPMI 1640 (Gibco, Carlsbad, CA) with 5% FCS.

### Adenovirus vectors

Ad vectors were produced on N52.E6 [45] cells. Vectors were released from infected cells by freeze-thaw with liquid nitrogen. Vectors were purified by one cesium chloride density step gradient, and one subsequent continuous cesium chloride density gradient. The lysis buffer and the gradients were supplemented with TCEP as reducing agent to prevent oxidation of the genetically introduced thiol-groups. Vectors were stored at −80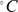 in an argon atmosphere as described previously [46]. Physical and infectious titers of unshielded vector particles were determined by a DNA-based slot-blot analysis [47]. Analysis was based on infection of A549 cells, removal of vector that had not entered the cells by washing with PBS and quantification of intracellular vector DNA. Table 1 summarizes the total and infectious titers of the vector preparations used in this work. Vector genome integrity was confirmed by restriction digestion of purified vector genomes and the presence of the cysteine residues was confirmed by sequencing. Vectors bearing a cysteine in hexon HVR5 were called AdHexCys.

### Synthesis of monomers

*N*-(2-Hydroxypropyl)methacrylamide (HPMA) was synthesized by a modified reaction of methacryloyl chloride with 1-aminopropan-2-ol in dichloromethane in the presence of sodium carbonate [48]. 3-(*N*-Methacryloylglycylglycyl)thiazolidine-2-thione (Ma-GlyGly-TT), 2-(trimethylammonio)ethyl methacrylate chloride (TMAEM) and 2-(pyridin-2-yldisulfanyl)-ethylamine, hydrochloride (Py-SS-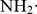
HCl) were prepared as described earlier [49]–[51].

### Synthesis of 2-Methyl-*N*-[2-(pyridine-2-yldisulfanyl)-ethyl]acrylamide (Ma-SS-Py)

Py-SS-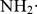
HCl (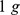, 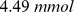) was suspended in dichloromethane (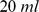) and triethylamine (Et_3_N) (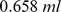, 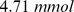) was added. Mixture was cooled to 0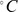 and methacryloyl chloride (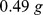, 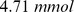) in dichloromethane (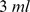) and Et_3_N (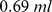, 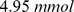) were added. Reaction mixture was stirred for 2 h at room temperature, then extracted 3 times with distilled water (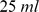) and dried with Na_2_SO_4_. Dichloromethane was evaporated and Ma-SS-Py was crystalized from ethyl acetate/hexane. Yield 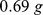. Elemental analysis: found/calcd. C = 51.94/51.98 H = 5.55/5.59 N = 11.01/10.94 S = 25.21/25.33

### Synthesis of HPMA copolymers

Polymer precursors P-GlyGly-TT and P-GlyGly-TT (TMAEM) were prepared by radical solution copolymerization. HPMA (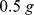, 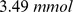), Ma-GlyGly-TT (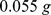, 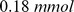) and 2,2′-azobis(2-methylbutyronitrile) (AIBN) (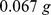) were dissolved in dimethyl sulfoxide (DMSO) (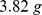), bubbled with nitrogen for 10 min. Copolymerization was carried out in sealed ampule at 60 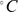 for 6 h. After copolymerization the copolymers were precipitated into an acetone diethyl ether (3∶1) mixture, filtered off, washed with acetone and diethyl ether and dried in vacuum. The P-GlyGly-TT (TMAEM) was prepared by the same procedure by using terpolymerization mixture consisting of HPMA (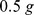, 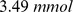), Ma-GlyGly-TT (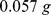, 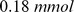), TMAEM (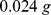, 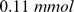) and AIBN (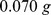) dissolved in DMSO (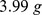).

The polymer conjugates P-GlyGly-mal (# 268) and P-GlyGly-mal (TMAEM) (# 269) were prepared by aminolysis of TT reactive groups in the P-GlyGly-TT and P-GlyGly-TT (TMAEM) precursors with *N*-(2-aminoethyl)maleimide trifluoroacetate in DMSO.

P-GlyGly-TT (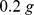, 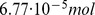 TT) was dissolved in DMSO (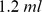) and *N*-(2-aminoethyl)maleimide trifluoroacetate (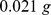, 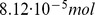) and ET_3_N (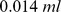, 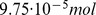) was added. Reaction mixture was stirred for 2 h at room temperature. Copolymer was precipitated into an acetone diethyl ether (3∶1) mixture and purified by column chromatography on Sephadex LH-20 in methanol. Yield of polymer conjugate P-GlyGly-mal was 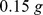. The polymer conjugates P-SS-Py (# 279 and # 280) differing in a content of pyridyldisulfide groups in the side chain were prepared by radical solution copolymerization of HPMA and Ma-SS-Py in DMSO at 60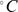 for 6 h. The polymer conjugates P-SS-Py (TMAEM) (# 281 and # 283) containing 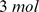
% of quaternary ammonio groups differing in a content of pyridyldisulfide groups in the side chain were prepared by radical solution terpolymerization of HPMA, Ma-SS-Py and TMAEM in DMSO at 60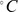 for 6 h.

### Characterization of HPMA copolymers

Number average molecular weight (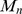), weight-average molecular weight (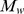), and polydispersity of polymer precursors and polymer conjugates were measured using size-exclusion chromatography (SEC) on a HPLC Shimadzu system equipped with UV, an Optilab® rEX differential refractometer and multi-angle light scattering DAWN® 8™ (Wyatt Technology, USA) detectors. For these experiments, 0.3 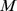 sodium acetate buffer pH = 6.5 and Superose 6 column were used. The content of thiazolidine-2-thione (TT) groups was determined spectrophotometrically on a Specord 205 (Jena Analytics) spectrophotometer (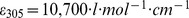; methanol). The content of pyridyldisulfide groups (PySS) in the polymer conjugates was determined by UV spectrophotometry after reaction with dithiothreitol [52]. The content of maleimidyl groups (mal) in polymer conjugates was determined by a modified Ellman€s assay, as a difference between concentration of cysteine in solution before and after reaction with maleimidyl groups of the polymer [53]. The characteristics of polymer conjugates are summarized in table 2.

### Chemical shielding of the vector surface

HPMA copolymer derivatives were used in a 20-fold molar excess over the genetically introduced cysteine residues. Immediately before shielding HPMA copolymer powder was dissolved in sterile-filtered, degassed and argon-saturated HEPES (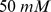; pH = 7.2). Vector preparation and HPMA copolymer solution were mixed and incubated over night in an argon atmosphere at room temperature. A minimum of 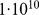 vector particles were shielded in a minimal volume of 20 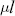.

### Western blot analysis


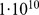
 vector particles were heated to 95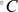 for 5 minutes in loading buffer that did or did not contain 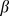-mercaptoethanol as indicated. After a sodium dodecyl sulfate polyacrylamide gel electrophoresis (SDS-PAGE) samples were transferred onto a nitrocellulose membrane. Primary antibody was mouse anti-hexon mAB (65H6, Abfrontier, Seoul, Korea) used 1∶5000. Secondary antibody was rabbit anti-mouse IgG (Sigma-Aldrich, A9044) used 1∶20,000. For the glutathione (L-glutathione reduced, Sigma-Aldrich, G4251) reduction analysis samples were preincubated with different concentrations of glutathione (0.05 to 10 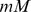) in HEPES (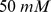; pH = 7.2) for 30 minutes at 37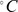. Then samples were mixed with loading buffer without 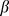-mercaptoethanol, heated to 95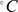 for 5 minutes and analyzed by Western blot.

### Transduction of A549 cells


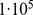
 cells were seeded in a 24-well plate in 1 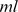 growth medium and cultivated over night. Cells were washed with PBS and 300 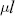 growth medium were added to each well. The cells were transduced with 200 pMOI of vector particles. After 2 hours of incubation, 1 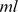 of growth medium was added to each well. The infected cells were incubated for 24 hours and then subjected to flow cytometry to analyze EGFP expression in a Beckman Coulter Gallios Flow Cytometer. The mean fluorescence intensity of unshielded AdHexCys was set to “1” and used to calculate the relative transduction efficiencies of the shielded vector particles.

### FX-mediated transduction of SKOV-3 cells


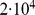
 SKOV-3 cells were seeded in a 96-well flat-bottom plate in 200 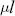 growth medium and cultivated over night. Cells were washed twice with PBS and serum-free growth medium supplemented with FX (CellSystems, Troisdorf, Germany) was added as indicated. Cells were transduced with 10,000 pMOI and incubated for 3 hours at 37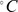. Afterwards cells were washed with 200 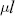 PBS twice and then given 200 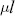 fresh growth medium supplemented with 5% fetal bovine serum, 1% penicillin-streptomycin-glutamine. 72 hours after transduction cells were subjected to flow cytometry to analyze EGFP expression in a Beckman Coulter Gallios Flow Cytometer. The mean fluorescence intensity of unshielded AdHexCys (supplemented with 50 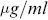 FX) was set to “1” and used to calculate the relative transduction efficiencies of the shielded vector particles.

### Labeling of vector particles with Alexa488-TFP and confocal laser scanning microscopy

Vector particles that were freshly shielded with HPMA copolymer derivatives the previous night were modified with a 20-fold molar excess of Alexa488-TFP (Alexa Fluor 488 Carboxylic Acid, 2,3,5,6-Tetrafluorophenyl Ester; Invitrogen, A-30005) over amine groups (18,000 per vector) for 2 hours at room temperature in an argon atmosphere in HEPES (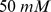, pH = 7.2). 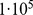 A549 cells were seeded in MatTek (Ashland, MA) poly-L-lysine-covered glass dishes and were cultivated over night. The next day, the cells were washed three times with ice cold PBS and 1 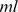 of ice cold medium was added. The cells were transduced with 10,000 pMOI of the differently shielded vector particles for 1 hour on ice. The cells were then washed three times with ice cold PBS and fresh ice cold medium was added, and the cells were kept on ice until they were placed in the incubation chamber of the confocal microscope. Live cell imaging was performed with a Carl Zeiss LSM 510 Meta confocal laser scanning microscopy (Carl Zeiss) equipped with a 63× water immersion objective (C-Apochromat 63×/1.20 W corr; Carl Zeiss, Jena, Germany) and a humidified CO_2_-controlled incubation chamber at 37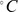.

### QPCR analysis

Quantitative PCR (QPCR) analysis was performed by amplification of the Ad5 E4 gene with a Stratagene 3005P QPCR machine using the Stratagene 2× Brilliant II SYBR Green QRT-PCR Master Mix Kit, 1-Step. To normalize for the cellular DNA content murine 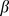-actin was used. To generate standard curves, liver DNA of naïve mice was spiked with pGS66 [45] containing the E4 gene. Primers: murine 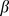-actin-sense (5′-caaggagtgcaagaacacag-3′), murine 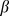-actin-antisense (5′-ctcaatacacactccaaggc-3′), E4-sense (5′-tagacgatccctactgtacg-3′), E4-antisense (5′-ccggacgtagtcatatttcc-3′). Forty cycles with the following thermal protocol were performed: melting (95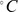; 30 seconds), annealing (60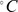; 30 seconds), and elongation (72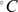; 30 seconds). For analysis, the E4-Ct-(dR) values were used.

### In vivo experiments

Female BALB/cAnNCrl (BALB/c) mice were obtained from an animal breeding colony (Charles River, Sulzfeld, Germany) and maintained in pathogen-free, individually ventilated cages. The animals were fed with sterilized diet for laboratory rodents (Ssniff, Soest, Germany) and used in experiments at 6–8 weeks of age. All experiments were approved by local authorities and were in accordance with local institutional guidelines. 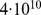 EGFP-expressing vector particles were injected i.v. into the tail vein of mice in a total volume of 200 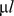 in HEPES (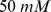, pH = 7.2). 72 hours after injection the mice were sacrificed by isoflurane (Forene, Abbott, Ludwigshafen, Germany) inhalation and livers were perfused with PBS. A part of the liver, spleen, kidney and lung was treated for histological sections and the rest of the organs was snap-frozen in liquid nitrogen and then stored at −80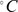 for DNA isolation (all organs) and fluorimetric analysis (liver only).

### Warfarin treatment

Four days and one day before vector injection, mice were injected subcutaneously with 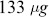 warfarin (Sigma-Aldrich, Steinheim, Germany) dissolved in 100 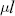 peanut oil to deplete them of vitamin K-dependent coagulation factors.

### Fluorimetric analysis of mouse liver homogenates


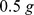
 extensively perfused and snap-frozen liver was homogenized in 1 ml homogenization buffer (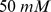 Tris, 1% NP-40, 0.25% sodium desoxycholate, 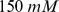 NaCl, 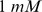 EDTA, in demineralized water, pH = 7.4, supplemented with Roche cOmplete EDTA-free Protease Inhibitor Cocktail) with a conical tissue grinder (Wheaton, Millville, NJ), transferred into a 1.5 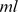 reaction tube and incubated for 10 minutes at room temperature. The samples were centrifuged for 10 minutes, 20,000 g, 4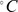. 500 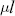 of the supernatant clear fraction were transferred into a new reaction tube and the rest was discarded. The sample was centrifuged for 10 minutes, 20,000 g, 4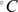. 200 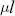 of the supernatant clear fraction were transferred into a new reaction tube and used for fluorimetry. The supernatant obtained was diluted 1∶500 to 1∶2,000 in homogenization buffer in a total volume of 2 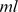 and used for fluorimetric analysis. Samples were analyzed in a quarz cuvette. An arc lamp was used as light source. Monochromatic light of 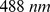 was obtained thereof using a computer controlled Spex 1680 0.22 m Double monochromator (Horiba Scientific). This was used for sample illumination in a Spex Fluorolog sample illuminator. Light emission was detected at 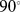. Another Spex 1680 0.22 m Double monochromator was used to obtain monochromatic light of 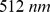 from the emitted spectrum. This was used for quantification with a thermoelectrically cooled photomultiplier diode (Hamamatsu R 928). Photon counts were averaged over a time of 10 seconds. SPEX Instruments SA. Inc., Spex dM3000 software version 3.32e was used for analysis.

### Histological analysis of mouse organs

Tissues were fixed in 2% PFA in PBS over night, in 30% sucrose in water over the following night, embedded in Tissue-Tek (Sakura, Zoeterwoude, The Netherlands) and stored at −80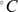. 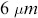 sections were made with a Leica-cryostat, put on silane covered glass slides and analyzed for EGFP expression or analyzed by immunofluorescence (see below). Sections were covered with fluorescent mounting medium (Dako, Copenhagen, Denmark) and analyzed with a Zeiss Axio Scope 2 plus fluorescence microscope.

### Immunohistological analysis of the spleen

Spleen sections were analyzed using anti-CD11c, anti-ER-TR7 and anti-MADCAM-1 antibodies. Sections were fixed for 10 min with ice-cold acetone (CD11c, MADCAM-1) or for 15 min with 4% PFA (ER-TR7). Sections were rinsed with PBS and blocked for 30 min with 10% normal goat serum (in PBS). Primary antibodies were diluted in PBS and incubated for 1 h at room temperature. The sections were rinsed with PBS twice for 10 min. Secondary antibodies were diluted in PBS-Tween 0.1% supplemented with 2% normal goat serum, and incubated for 1 h at room temperature. Sections were rinsed with PBS twice for 10 min. Sections were covered with fluorescence mounting medium. Pictures were taken with a Zeiss Axioskop 2 plus fluorescent microscope. Exposure time of images was 2,000 ms. Hamster anti-mouse CD11c mAB (1∶100; BD, 550283, Franklin Lakes, NJ, USA) was used with the secondary AB goat anti-hamster AlexaFluor546 (1∶500; Invitrogen Ltd., Paisley, GB). Rat anti-mouse ER-TR7 mAB (1∶33; Abcam, ab51824, Cambridge, GB) and Rat anti-mouse MADCAM-1 mAB (1∶33; eBioscience, Inc., 14-5997-85, San Diego, CA, USA) were used with the secondary AB goat anti-rat AlexaFluor555 (1∶500; Invitrogen Ltd., Paisley, GB).

### Blood circulation kinetics

Mice were anesthesized by i.p. injection of Ketamin/Xylazin. 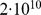 vector particles were injected i.v. into the tail vein of mice in a total volume of 200 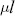 in HEPES (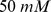, pH = 7.2). Shortly after injection (2, 4, 6, 10 and 20 min) 20 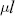 of blood were taken with a heparinized glass capillary by scoring the tail with a scalpel. Mice were culled after 20 min. Total DNA was extracted with the GenElute Mammalian Genomic DNA Miniprep Kit (Sigma-Aldrich) according to the manufacturer's instructions. Ad-genome content was analyzed by quantitative PCR.

### Statistics

The significance of the data was determined by using the Wilcoxon-Mann-Whitney-Test using RStudio-software Version 0.96.228.

## Supporting Information

Figure S1
**HPMA copolymers can be released by glutathione.** 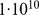 vector particles were shielded with HPMA copolymers and analyzed by Western blot analysis using a monoclonal anti-hexon antibody. Before loading, the samples were pre-incubated with different concentrations of glutathione (as indicated) for 30 min. at 37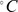. Loading buffer did not contain 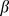-mercaptoethanol. A: AdHexCys was shielded with HPMA copolymer # 279. B: AdHexCys was shielded with HPMA copolymer # 280. C: AdHexCys was shielded with HPMA copolymer # 283. The “+ Polymer-number” indicates a shielding of AdHexCys with the respective HPMA copolymer.(PNG)
