# Supplementary figures and images for: Traceless Bioresponsive Shielding of Adenovirus Hexon with HPMA Copolymers Maintains Transduction Capacity In Vitro and In Vivo
Source: PLoS One. 2014 Jan 27;9(1):e82716. doi: 10.1371/journal.pone.0082716 (PMC3903484; doi:10.1371/journal.pone.0082716)

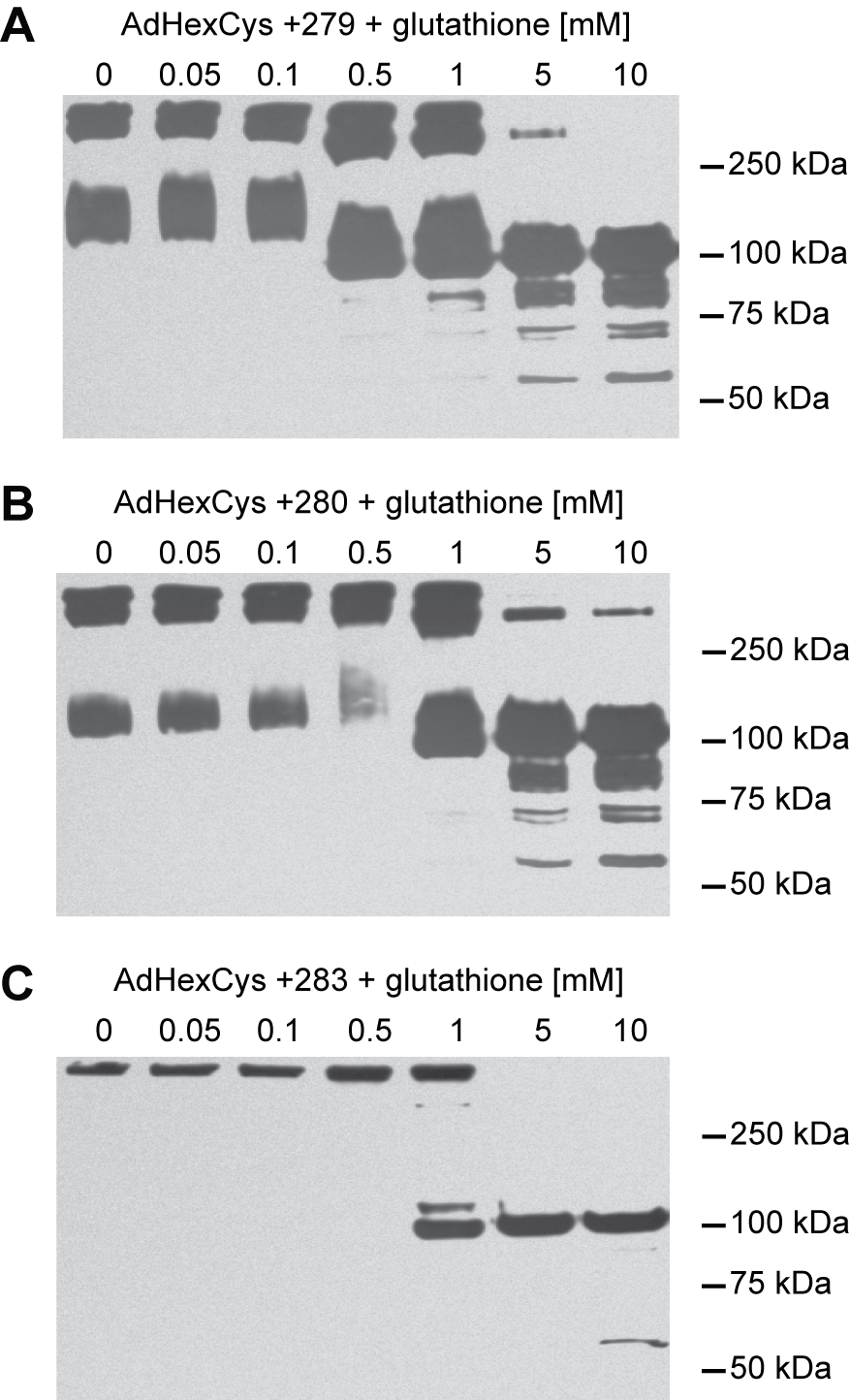

Supplement: Figure S1 — HPMA copolymers can be released by glutathione. vector particles were shielded with HPMA copolymers and analyzed by Western blot analysis using a monoclonal anti-hexon antibody. Before loading, the samples were pre-incubated with different concentrations of glutathione (as indicated) for 30 min. at 37. Loading buffer did not contain -mercaptoethanol. A: AdHexCys was shielded with HPMA copolymer # 279. B: AdHexCys was shielded with HPMA copolymer # 280. C: AdHexCys was shielded with HPMA copolymer # 283. The “+ Polymer-number” indicates a shielding of AdHexCys with the respective HPMA copolymer. (PNG) [file pone.0082716.s001.png]
